# Prognostic Power of Pulmonary Arterial Compliance Is Boosted by a Hemodynamic Unloading Test With Glyceryl Trinitrate in Heart Failure Patients With Post-capillary Pulmonary Hypertension

**DOI:** 10.3389/fcvm.2022.838898

**Published:** 2022-03-31

**Authors:** Andreas J. Rieth, Dimitri Grün, Georgios Zarogiannis, Steffen D. Kriechbaum, Sebastian Wolter, Manuel J. Richter, Khodr Tello, Ulrich Krüger, Veselin Mitrovic, Stephan Rosenkranz, Christian W. Hamm, Till Keller

**Affiliations:** ^1^Department of Cardiology, Kerckhoff-Klinik, Bad Nauheim, Germany; ^2^German Center for Cardiovascular Research (DZHK), Partner Site RheinMain, Frankfurt am Main, Germany; ^3^Department of Internal Medicine I, Cardiology, Justus-Liebig-University Giessen, Giessen, Germany; ^4^Department of Cardiology, Evangelisches Klinikum Niederrhein, Duisburg, Germany; ^5^Department of Pneumology, Kerckhoff-Klinik, Bad Nauheim, Germany; ^6^Department of Internal Medicine, Universities of Giessen and Marburg Lung Center (UGMLC), Member of the German Center for Lung Research (DZL), Justus Liebig University Giessen, Giessen, Germany; ^7^Clinic III for Internal Medicine, Department of Cardiology, Heart Center at the University of Cologne and Cologne Cardiovascular Research Center (CCRC), University of Cologne, Cologne, Germany

**Keywords:** pulmonary arterial compliance, glyceryl trinitrate (GTN), vasoreactivity testing, post-capillary pulmonary hypertension, hemodynamics, prognosis

## Abstract

**Background:**

Pulmonary hypertension (PH) is an established risk factor in patients with heart failure (HF). However, right heart catheterisation (RHC) and vasoreactivity testing (VRT) are not routinely recommended in these patients.

**Methods:**

The primary objective of the present study was to explore the impact of VRT using sublingual glyceryl trinitrate (GTN) on transplant/ventricular assist device-free survival in HF patients with post-capillary PH. RHC parameters were correlated retrospectively with the primary outcome.

**Results:**

The cohort comprised 154 HF patients with post-capillary PH undergoing RHC with GTN-VRT at a tertiary heart failure centre. Multiple parameters were associated with survival. After adjustment for established prognosis-relevant clinical variables from the MAGGIC Score, variables with the most relevant odds ratios (OR) obtained after GTN-VRT were: calculated effective pulmonary arterial (PA) elastance (adjusted OR 2.26, 95%CI 1.30–3.92; *p* = 0.004), PA compliance (PAC-GTN; adjusted OR 0.45, 95%CI 0.25–0.80; *p* = 0.006), and total pulmonary resistance (adjusted OR 2.29, 95%CI 1.34–3.93; *p* = 0.003). Forest plot analysis including these three variables as well as PAC at baseline, delta PAC, and the presence of combined post- and pre-capillary PH revealed prognostic superiority of PAC-GTN, which was confirmed by Kaplan-Meier analysis.

**Conclusions:**

In our cohort of symptomatic HF patients with post-capillary PH, improved PAC after administration of GTN was associated with survival independent of established hemodynamic and clinical risk factors. VRT using GTN may be better described as unloading test due to GTN's complex effects on the circulation. This could be used for advanced prognostication and should be investigated in further studies.

## Graphical Abstract

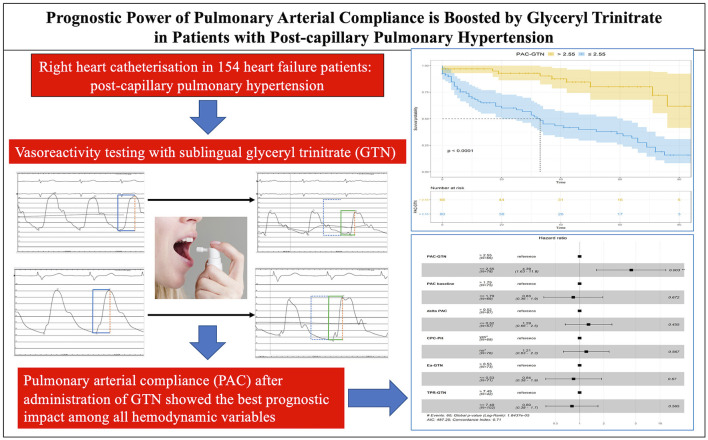

## Introduction

Post-capillary pulmonary hypertension (PH) is an established risk factor in patients with left heart failure (LHF), and those with advanced pulmonary vascular remodeling are known to have worse prognosis than those without. Increased vascular stiffness as a consequence of specific changes in the pulmonary vasculature leads to enhanced right ventricular afterload and right heart failure, which drives mortality in these patients (1). However, there is an ongoing debate concerning which hemodynamic parameters best mirror the extent of fixed pulmonary arterial stiffening and thus best identify patients with poor prognosis. Pulmonary vascular resistance (PVR) and pulmonary arterial compliance (PAC) seem to be the strongest prognostic indices in patients with PH associated with LHF (2–5). However, current guidelines define the subgroup of post-capillary PH with worse prognosis using a combination of PVR >3 wood units (WU) and/or diastolic pressure gradient (DPG) ≥7 mmHg, denoted combined post- and pre-capillary pulmonary hypertension (CpcPH), in contrast to isolated post-capillary pulmonary hypertension (Ipc-PH), which is associated with a slightly better prognosis (6). Recently, a modified classification was proposed, with PVR ≥ 3 WU as a single indicator of CpcPH (7).

Isolated hemodynamic measurements are subject to significant spontaneous variations. The use of serial measurements, e.g., after acute vasoreactivity testing (VRT), likely improves hemodynamic prognostication (8, 9). However, in patients with post-capillary PH, VRT is recommended only as a part of the evaluation for heart transplantation, with fixed PH being a potential contraindication because of a particularly high risk for postoperative right heart failure (6, 10). Beyond this scenario, information gained from an acute vasodilator challenge is of uncertain clinical significance, possibly owing to the heterogeneity of vasodilators used for testing in LHF patients, missing standard protocol and the lack of studies on prognostic implications (11). Despite these facts, VRT is part of the standard hemodynamic workup of patients with LHF in several heart failure centres; according to local customs, sublingual glyceryl trinitrate (GTN) may be used as vasodilator (12). GTN as an arterial and venous vasodilator may be advantageous in LHF patients compared with selective pulmonary vasodilators such as inhaled nitric oxide or iloprost, because the latter may lead to an increase of left ventricular filling pressures and pulmonary edema (11).

The purpose of the present study was to explore the association of VRT results using sublingual GTN with outcomes in LHF patients with post-capillary PH. We hypothesized that application of GTN could provide incremental prognostic information by unmasking substantial pulmonary vascular disease.

## Materials and Methods

### Study Population

The study cohort comprised the ongoing, prospectively recruiting Kerckhoff-Klinik HF Registry. The dataset included 154 consecutive patients registered from 10/2009 to 02/2016 who were assessed for heart failure by right heart catheterization (RHC) that included vasoreactivity testing with GTN. Inpatients (*n* = 85, 55%) were hospitalized because of worsening heart failure (31.8%), acutely decompensated heart failure (28.2%), diagnostic workup for evaluation of dyspnea (23.5%), and suspected pulmonary hypertension (16.5%). Inclusion criteria were a diagnosis of LHF with preserved or reduced left ventricular (LV) function according to current guidelines, availability of sufficient hemodynamic data, mean pulmonary artery pressure (mPAP) >20 mmHg and pulmonary artery wedge pressure (PAWP) >15 mmHg. PAWP between 10 and 15 mmHg at rest was accepted in a few cases (*n* = 12) if PAWP increased >25 mmHg during exercise or if clear features of left heart disease such as LV hypertrophy, reduced LV function, and/or significant left atrial enlargement were present. All patients included underwent guideline-compliant treatment for HF excluding PH targeted drugs. Exclusion criteria were loss to follow-up (at least one follow-up visit was required apart from the evaluation visit with RHC), severe heart valve stenosis, congenital heart defects, and constrictive pericarditis (Supplementary Figure 1).

The investigation conforms with the principles outlined in the Declaration of Helsinki. All patients enrolled in the registry gave written informed consent. Data collection and analyses were approved by the ethics committee of the Faculty of Medicine at the University of Giessen (approval no. 220/15; 26 January, 2016).

### Outcomes

The primary outcome measure was defined as survival free from heart transplantation (HTX) and left ventricular assist device (LVAD) implantation. Survival data were obtained through clinically indicated follow-up visits or telephone contact.

### Basic Diagnostics

All patients underwent transthoracic echocardiography according to recommendations of the respective guidelines as part of the clinical work-up with determination of left ventricular ejection fraction (LVEF), tricuspid annular plane systolic excursion (TAPSE), estimated systolic pulmonary artery pressure (sPAP), and valve assessment. Baseline laboratory examinations including N-terminal brain natriuretic peptide (NT-proBNP) and estimated glomerular filtration rate (eGFR), based on serum creatinine, were carried out by the respective in-house central laboratory as part of the clinical routine care.

### Hemodynamic Assessment and Vasodilator Challenge

RHC was performed in recompensated, stable patients under local anesthesia with insertion of a Swan-Ganz catheter (7F Thermodilution Catheter, Biosensors International, Singapore or Edwards Lifesciences) via the internal jugular vein or a cubital vein as described previously (13). The zero reference level for the pressure transducer was placed at the mid-thoracic level as recommended for the supine position, and all pulmonary pressures were taken at end-expiration and averaged over a minimum of 3 cardiac cycles. Baseline measurements were repeated after 20 min of rest. Those patients able to perform bicycle exercise (*n* = 90, 58%) were measured again during exercise. Instead of bicycle exercise, volume challenge (passive leg raise) was performed in 17 patients (11%) for additional measurements. After exercise / volume challenge, return to resting values was required for continuation of the examination. If mPAP was >20 mmHg and systolic blood pressure >100 mmHg, GTN was administered sublingually at an initial standard dose of 1.2 mg. GTN administration was repeated according to in-house standard operating procedures. The waiting time for repetition of measurements was a minimum of 5 min. A definition of positive response to GTN challenge was not determined in advance.

Cardiac output (CO) was determined by the thermodilution (TD) technique. The calculated parameters were: stroke volume (SV = CO/heart rate); total pulmonary resistance (TPR = mPAP/CO); pulmonary arterial (PA) pulse pressure (PP = sPAP–dPAP); PA compliance (PAC = SV/PP); effective PA elastance (Ea = (1.65 x mPAP−7.79)/SV) (14); PA pulsatility index (PAPi = PP/RAP); mean right ventricular (RV) power (mPAPxCO); total RV power (1.3 x mean RV power); oscillatory RV power (total–mean RV power) (15). Measurements before GTN administration are referred to as “baseline,” after administration as “-GTN,” and the difference between the two as “delta.”

### Statistical Analysis

Data are expressed as mean ± standard deviation or median [interquartile range] for normally or non-normally distributed parameters, respectively. Adherence to a Gaussian distribution was determined using the Shapiro test.

For independent samples, comparison was made with the Independent-Samples Kruskal-Wallis test for non-normally distributed parameters, the Student *t* test for normally distributed parameters, and Fisher's exact test for categorical parameters, as appropriate. For dependent samples, the paired *t* test was used for normally distributed parameters, and otherwise the Wilcoxon signed rank test.

We selected variables with the best predictive value for transplant/LVAD-free survival based on their ability to improve the predictive value of the MAGGIC score variables, an established score for risk prediction in patients with heart failure (16–18). Odds ratios (OR) were calculated based on the z-scores of each variable and are referenced as risk per standard deviation. Receiver operator characteristic (ROC) analysis with the calculated area under the curve (AUC) were used to describe an association of a variable with survival. Based on the results of ROC analysis, optimal cutoff values for prediction of mortality were calculated using the Youden index. Furthermore, based on these cutoff values multivariable Cox proportional hazards models and the Kaplan-Meier method were used for survival analyses. *P* <0.05 was considered statistically significant. Statistical analyses were performed using either R version 3.6.0 (survival package 3.2-3, survminer package 0.4.8) or GraphPad Prism version 8.4.3 (471).

## Results

### Baseline Characteristics

This mono-centric analysis included 154 patients (39% female). Median age was 71 (IQR 62–76) years and 75% of the patients presented with symptoms according to NYHA class III. NT-proBNP levels [median 1890 (IQR 973-4182) pg/ml] were markedly elevated. Median LVEF was 45 [25–55]%, and the median TAPSE was 15 mm [12–19]. Classification according to heart failure type was as follows: 74 patients with preserved EF (≥50%), HFpEF; 12 with mid range reduced EF (40–49%), HFmrEF; and 68 with reduced EF (<40%), HFrEF). Duration of heart failure ≥ 18 months was present in 27% of HFpEF, 75% of HFmrEF and 79% of HFrEF patients. In HFpEF patients, 84% had a history of hypertension, 36% had coronary artery disease, 28% suffered from diabetes, 81% had atrial fibrillation, and 2 patients had hypertrophic cardiomyopathy. In HFmrEF patients, the etiology of HF was hypertensive in 33% and ischemic in 25%; 2 patients had dilated and 3 valvular cardiomyopathy. In HFrEF patients, 54% had ischemic etiology, and 34% had dilated cardiomyopathy, 7% valvular cardiomyopathy, 3% hypertensive cardiomyopathy; 1 patient had congenital heart disease.

Nearly all patients (94%) were treated with diuretics; guideline-directed medical therapy was present as indicated. Patients had a high frequency of atrial fibrillation/flutter (73%) and device therapy (51%). Baseline characteristics, also stratified by our PAC-GTN cutoff, are provided in Table 1.

**Table 1:** Baseline characteristics.

	**Data**	**All**	**PAC-GTN > 2.55**	**PAC-GTN ≤2.55**	*p*-Value^*^
	**availability**	***n* = 154**	***n* = 66^§^**	***n* = 82^§^**	
All-cause mortality/HTX/LVAD Median Survival, months	154/154	66 (43)		33	<0.001
GTN dose, mg	151/154	2.4 [1.6–3.2]	2.4 [1.2–3.2]	2.4 [1.6–3.2]	0.324^a^
Female sex^#^	154/154	60 (39)	28 (42)	29 (35)	0.400
Age^#^, years	154/154	71.0 [62.0–76.0]	73.0 [63.3–77.0]	68.0 [59.0–75.0]	0.095^a^
Body mass index^#^, kg/m^2^	154/154	28.9 [25.3–33.4]	31.0 [27.2–34.3]	27.8 [24.1–32.4]	0.003^a^
Smoker^#^ (current or within last 6 months)	154/154	12 (7.8)	6 (9.1)	6 (7.3)	0.767
Hypertension^#^	154/154	125 (81.2)	55 (83.3)	65 (79.3)	0.673
Coronary artery disease	154/154	69 (44.8)	26 (39.4)	42 (51.2)	0.185
Atrial fibrillation/flutter	154/154	113 (73.4)	45 (68.2)	62 (75.6)	0.358
Diabetes mellitus^#^	154/154	56 (36.4)	19 (28.8)	35 (42.7)	0.089
Diagnosis of CHF ≥ 18 months^#^	154/154	83 (53.9)	29 (43.9)	53 (64.6)	0.013
COPD^#^	154/154	25 (16.2)	16 (24.2)	9 (11.0)	0.046
Device therapy (ICD or pacemaker)	154/154	78 (50.7)	23 (34.9)	54 (65.9)	<0.001
Aldosterone blocker use	153/154	82 (53.6)	28 (42.4)	48 (59.3)	0.069
ß-Blocker use^#^	153/154	132 (86.3)	59 (89.4)	69 (85.2)	0.470
ACE inhibitor/ARB use^#^	153/154	136 (88.9)	57 (86.4)	73 (90.1)	0.624
Calcium channel blocker use	153/154	27 (17.7)	12 (18.2)	13 (16.1)	0.826
Cardiac glycoside use	153/154	34 (22.2)	8 (12.1)	26 (32.1)	0.006
Diuretic use	153/154	144 (94.1)	63 (95.5)	76 (93.8)	0.732
**NYHA class** ^**#**^	153/154				
I		4 (2.6)	3 (4.5)	1 (1.2)	
II		27 (17.5)	13 (19.7)	10 (12.2)	
III		114 (74.0)	47 (71.2)	65 (79.3)	0.337
IV		8 (5.2)	2 (3.0)	6 (7.3)	
V'O_2_ peak, ml/min/kg	78/154	11.0 [9.6–13.2]	11.9 [10.7–14.3]	9.9 [8.9–12.5]	0.002^a^
Maximum workload, W	78/154	50 [40–60]	60 [40–70]	40 [30–50]	<0.001^a^
GFR^#^, ml/min/1.73 m^2^	145/154	62.0 [48.1–81.0]	68.6 [53.8–94.7]	58 [44.5–72.0]	0.009^a^
Urea, mg/dl	126/154	54.0 [38.0–78.5]	42.0 [34.8–66.8]	59.0 [44.0–81.0]	0.005^a^
Sodium, mmol/l	145/154	139 [136–141]	140 [138–141]	138 [135–140]	0.008^a^
NT-proBNP, pg/ml	141/154	1,890 [973–4182]	1,137 [602–2405]	3,281 [1732–5165]	<0.001^a^
LVEF^#^, %	154/154	45 [25–55]	55 [39–55]	35 [20–55]	<0.001^a^
TAPSE, mm	109/154	15 [12–19]	18 [16–20]	13 [11–16]	<0.001^a^
RVSP, mmHg	131/154	54 [43–66]	49 [39–60]	58 [47–70]	0.004^a^

*Data are displayed as count (percentage), or median [interquartile range] except where otherwise indicated. GTN, glycerol trinitrate; HTX, heart transplantation; LVAD, left ventricular assist device; CHF, chronic heart failure; COPD, chronic obstructive pulmonary disease; ICD, implantable cardiac defibrillator; ACE, angiotensin-converting enzyme; ARB, angiotensin receptor blocker; NYHA, New York Heart Association; V'O_2_ peak, oxygen uptake measured by cardiopulmonary exercise testing; GFR, glomerular filtration rate; NT-proBNP, N-terminal fragment of pro-brain natriuretic peptide; LVEF, left ventricular ejection fraction; TAPSE, tricuspid annular plane systolic excursion; RVSP, right ventricular systolic pressure derived from tricuspid regurgitation velocity*.

* *PAC-GTN > 2.55 vs. PAC-GTN ≤ 2.55*.

§ *Stroke volume and thus PAC after GTN administration was not available in 6 patients*.

a *Mann–Whitney U test. All categorical variables were compared using Fisher's exact test*.

# *Tagging of the variables used for the MAGGIC-Score*.

### Effects of GTN Administration

**Figure 1:**
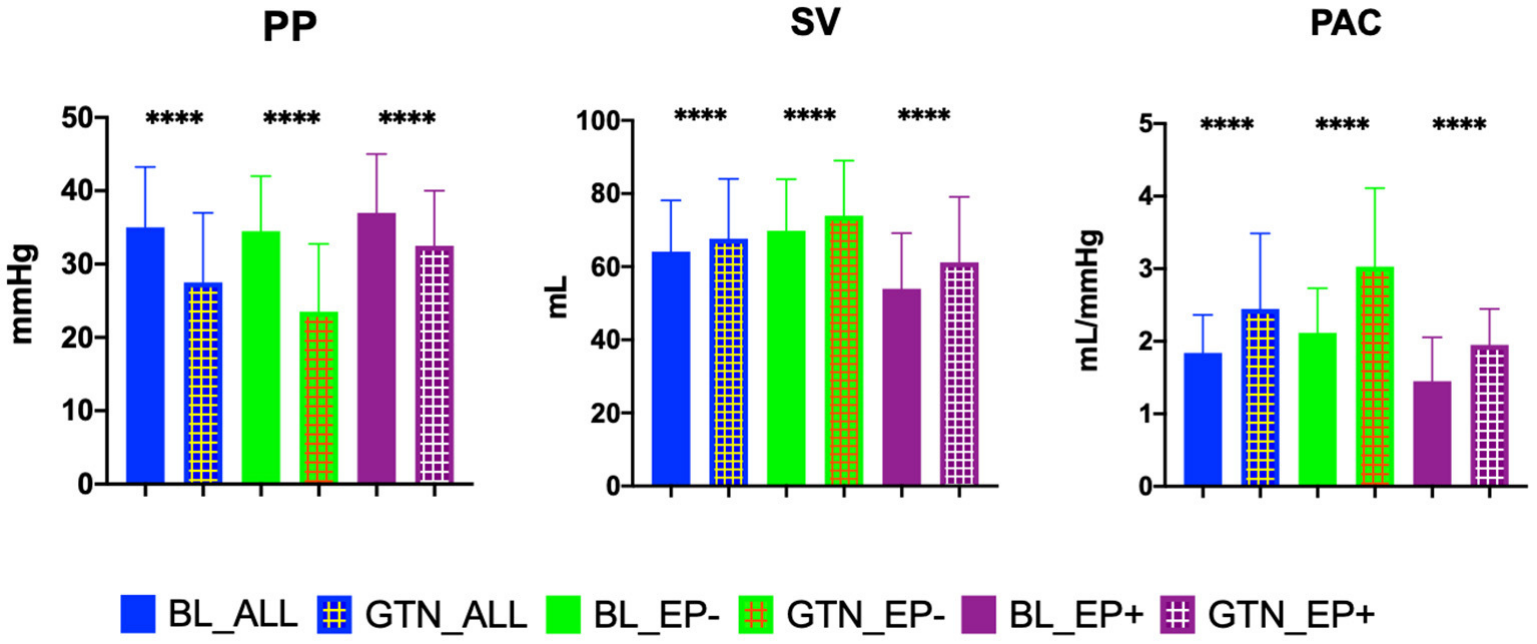
Key hemodynamic parameters at baseline and after glyceryl trinitrate (GTN) application in all patients, in patients who did not reach the endpoint transplant/LVAD free survival (EP-), and in patients reaching the endpoint (EP+). PP, pulse pressure; SV, stroke volume; PAC, pulmonary arterial compliance; BL, baseline. ****P <0.00001.

Median administered GTN dose was 2.4 (IQR 1.6-3.2) mg. GTN vasodilator challenge led to a significant change in most hemodynamic parameters, except heart rate and the PVR/SVR ratio (Supplementary Table 1*)*. There were no significant side effects of GTN, especially no serious hypotension. Hemodynamics before and after GTN administration, stratified by our PAC-GTN cutoff, are provided in Table 2. Comparing survivors with those who died or underwent HTX/LVAD, survivors showed a smaller increase in SV (median +3.11 vs. +6.52 ml) but a markedly larger decrease in PP (−9.0 vs. −3.5 mmHg) and a subsequent larger increase (improvement) in PAC (+0.89 vs. +0.30 ml/mmHg) than those meeting the end point (Figure 1). Furthermore, there were numerous differences in response to GTN between these groups.We compared the hemodynamic parameters before and after GTN administration in different types of LHF (HFpEF, HFmrEF and HFrEF). There were no significant differences in baseline PAP. After GTN administration, patients with HFpEF showed the lowest increase in CO and decrease of Ea and systemic vascular resistance, but the largest reduction of PP. Patients with HFrEF had the lowest increase of PAC and decrease of PP, and also lowest fall in systolic blood pressure (Supplementary Table 2).

**Table 2:** Hemodynamics at baseline and after GTN administration.

	**BASELINE**					**GTN**				
**Data availability**		**PAC-GTN > 2.55**		**PAC-GTN ≤2.55**	*p*-Value^*^		**PAC-GTN > 2.55**		**PAC-GTN ≤2.55**	*p*-Value^*^
**(D.a.)**	**D.a**.	***n = 66^§^***	**D.a**.	***n = 82^§^***		**D.a**.	***n = 66^§^***	**D.a**.	***n = 82^§^***	
Systolic BP, mmHg	66	134.0 (±25.0)	82	119 [106-136]	0.006^a^	66	124.0 (±22.0)	82	112 [101-128]	0.003^a^
Mean BP, mmHg	66	93 [84–106]	81	88 [80–97]	0.016^a^	65	88.0 (±14.0)	81	82.0 (±12.0)	0.003^b^
Heart rate, beats/min	66	67.0 (±10.0)	82	70.0 (±9.5)	0.098^b^	66	67 [61–73]	82	69 [62–75]	0.286^a^
PAWP, mmHg	66	21.0 [18–24]	82	26.0 (±5.0)	<0.0001^a^	66	13.0 [9.0–18.0]	82	20.0 [16.0–24.0]	<0.0001^a^
sPAP, mmHg	66	51.0 (±11.0)	82	68.0 [56–73]	<0.0001^a^	66	35.0 [29–45]	82	55.0 (±13.0)	<0.0001^a^
mPAP, mmHg	66	33.0 (±6.3)	82	41.0 (±6.7)	<0.0001^b^	66	23.0 (±6.0)	82	32.0 (±7.3)	<0.0001^b^
dPAP, mmHg	66	20.0 (±4.8)	82	25.0 (±5.6)	<0.0001^b^	66	*15.0 (±4.7)*	82	19.0 (±5.8)	<0.0001^b^
PP, mmHg	66	30.0 [25.0–37.0]	82	40.0 [32.0–47.0]	<0.0001^a^	66	20.0 [17.0–25.0]	82	36.0 [29.0–42.0]	<0.0001^a^
RAP, mmHg	66	11.0 [7.0–13.0]	82	12.0 [9.0–17.0]	0.009^a^	63	7.0 [5.0–10.0]	71	9.0 (±4.9)	0.056^a^
TPG, mmHg	66	11.0 [8.0–14.0]	81	15.0 [11.0–19.0]	<0.0001^a^	66	9.6 (±3.7)	81	14.0 (±5.3)	<0.0001^b^
DPG, mmHg	66	−0.92 (±4.1)	81	−1.0 [−4.0-3.0]	0.572^a^	66	1.4 (±3.9)	81	0.28 (±5.3)	0.169^b^
CO-TD, l/min	66	5.0 [4.1–5.9]	82	3.7 [3.1–4.5]	<0.0001^a^	66	5.4 (±1.3)	82	4.2 [3.5–5.1]	<0.0001^a^
CI-TD, l/min/m^2^	66	2.5 [2.2–2.8]	82	2.0 [1.7–2.3]	<0.0001^a^	66	2.7 (±0.63)	82	2.2 [2.0–2.6]	<0.0001^a^
SV-TD, mL	66	72.0 [62.0–92.0]	82	54.0 [45.0–69.0]	<0.0001^a^	66	77.0 [64.0–91.0]	82	61.0 [49.0–77.0]	<0.0001^a^
PVR, WU	66	2.3 (±0.9)	81	3.6 [2.9–5.4]	<0.0001^a^	66	1.9 (±0.74)	81	3.0 [2.2–4.2]	<0.0001^a^
SVR, WU	66	19.0 [16.0–22.0]	82	24 (±6.5)	<0.0001^a^	66	17.0 [13.0–20.0]	81	20 (±5.3)	0.002^a^
PVR/SVR	66	0.12 [0.08–0.16]	80	0.16 [0.13–0.22]	<0.0001^a^	65	0.11 (±0.05)	80	0.17 (±0.07)	<0.0001^b^
TPR, WU	66	6.6 (±1.5)	82	11.0 [8.8–13.0]	<0.0001^a^	66	4.5 (±1.2)	82	7.6 [6.0–8.9]	<0.0001^a^
PAC, mL/mmHg	66	2.4 [2.1–3.0]	82	1.4 [1.1–1.7]	<0.0001^a^	66	3.5 [3.0–4.5]	82	1.9 [1.4–2.2]	<0.0001^a^
Ea, mmHg/mL	66	0.63 (±0.18)	82	1.0 [0.89–1.4]	<0.0001^a^	66	0.40 (±0.14)	82	0.73 [0.57–0.87]	<0.0001^a^
PAPi	66	3.0 [2.2–4.0]	82	3.3 [2.2–4.8]	0.263^a^	63	3.0 [2.3-4.2]	71	4.3 [3.2–6.3]	<0.0001^a^
RAP/PAWP	66	0.53 [0.40–0.64]	80	0.53 [0.36–0.62]	0.906^a^	63	0.56 [0.42–0.65]	74	0.43 (±0.21)	0.006^a^
RV power_oscill_, W	66	0.11 [0.08–0.13]	82	0.10 [0.08–0.13]	0.524^a^	66	0.08 [0.06–0.11]	82	0.09 [0.07–0.12]	0.093^a^

*Data are displayed as median [interquartile range] or mean (± standard deviation) except where otherwise indicated. GTN, glycerol trinitrate; BP, blood pressure; PAWP, pulmonary arterial wedge pressure; sPAP, systolic pulmonary arterial pressure;x mPAP, mean PAP; dPAP, diastolic PAP; PP, pulse pressure; RAP: mean right atrial pressure; TPG, transpulmonary gradient; DPG, diastolic pulmonary gradient; CO, cardiac output; TD, Thermodilution method; CI, cardiac index; SV, stroke volume; PVR, pulmonary vascular resistance; WU, Wood units; SVR, systemic vascular resistance; TPR, total pulmonary resistance; PAC, pulmonary arterial compliance; Ea, pulmonary effective arterial elastance (calculated); PAPi, pulmonary artery pulsatility index; RV, right ventricle; oscill, oscillatory; W, watt*.

* *PAC-GTN > 2.55 vs. PAC-GTN ≤ 2.55*.

§ *Stroke volume and thus PAC after GTN administration was not available in 6 patients*.

a *Mann–Whitney U test*.

b *Student's t-test*.

### Association of GTN-Dependent Hemodynamics and Outcome

The median follow-up was 30 [8–57] months in our cohort. Within this period 62 (40.3%) patients died, 3 (1.9%) underwent HTX, and 1 (0.6%) underwent LVAD implantation. Overall survival free from HTX and LVAD implantation was 57.2%.

Univariate regression analysis revealed multiple associations between hemodynamic parameters and survival, both at baseline and post-GTN testing. Variables with the best ability to improve the predictive value of the MAGGIC score variables were selected. The components of the MAGGIC-Score are tagged within Table 1, and data on the univariate associations and the MAGGIC score are given in Supplementary Table 3. The three hemodynamic measures showing the strongest association (lowest adjusted *p*-values in combination with highest AUC) with outcome were Ea-GTN, TPR-GTN, and PAC-GTN, all after GTN challenge.

The areas under the curve (AUC) in the ROC analysis of these parameters (adjusted for the MAGGIC score variables) to discriminate patients with poor outcome were 0.89 (95% CI 0.83-0.95) for Ea-GTN, 0.89 (0.84–0.95) for TPR-GTN, and 0.89 (0.84–0.95) for PAC-GTN. The respective odds ratios (OR) adjusted for the MAGGIC score variables were 2.26 (1.30–3.92) per SD increase (*p* = 0.004) for Ea-GTN, 2.29 (1.34–3.93) per SD increase (*p* = 0.003) for TPR-GTN, and 0.45 (0.25–0.80) per SD increase (*p* = 0.006) for PAC-GTN.

**Figure 2:**
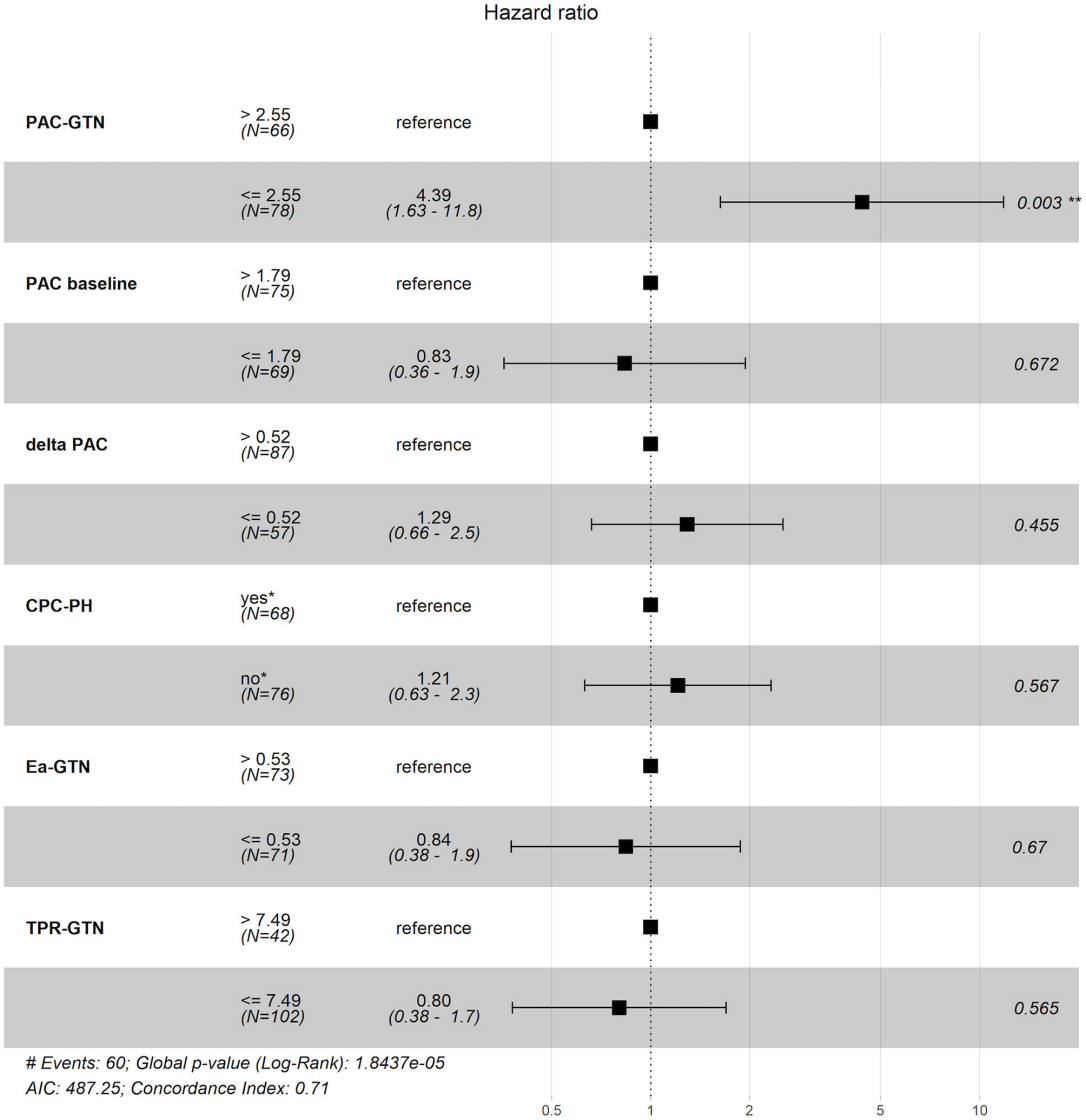
Forest plot of different hemodynamic variables and their impact on prognosis. PAC, pulmonary arterial compliance; CpcPH, combined post- and pre-capillary pulmonary hypertension (“yes,” if pulmonary vascular resistance >3 wood units and/or diastolic pulmonary gradient ≥7 mmHg); GTN, glyceryl trinitrate; Ea, pulmonary effective arterial elastance (calculated); TPR, total pulmonary resistance.

Optimal cut-off values for mortality, calculated using the Youden index, were 0.53 mmHg/ml for Ea-GTN, 7.49 WU for TPR-GTN and 2.55 ml/mmHg for PAC-GTN. The predictive value of these derived cut-off values was compared with the predictive information of the difference between baseline PAC and PAC-GTN (delta PAC), and established parameters such as PAC (baseline) and the presence of CpcPH (defined by PVR >3 WU and/or DPG ≥ 7 mmHg). In this multivariable analysis, considering several clinical important factors, PAC-GTN was the only independent significant factor associated with survival (Figure 2).

Correlation analyses were performed additionally to demonstrate independence of PAC-GTN related to known risk markers. In patients with PAC-GTN >2.55 ml/mmHg, there were the following correlations of PAC-GTN: vs. NT-pro BNP: *r* = −0.10; vs. LVEF: *r* = 0.08; vs. TAPSE: *r* = 0.17 (all *p*-values > 0.05); and in patients with PAC-GTN ≤ 2.55 ml/mmHg: vs. NT-pro BNP: *r* = −0.16; vs. LVEF: *r* = −0.09; vs. TAPSE: *r* = 0.22; all *p*-values > 0.05.

**Figure 3:**
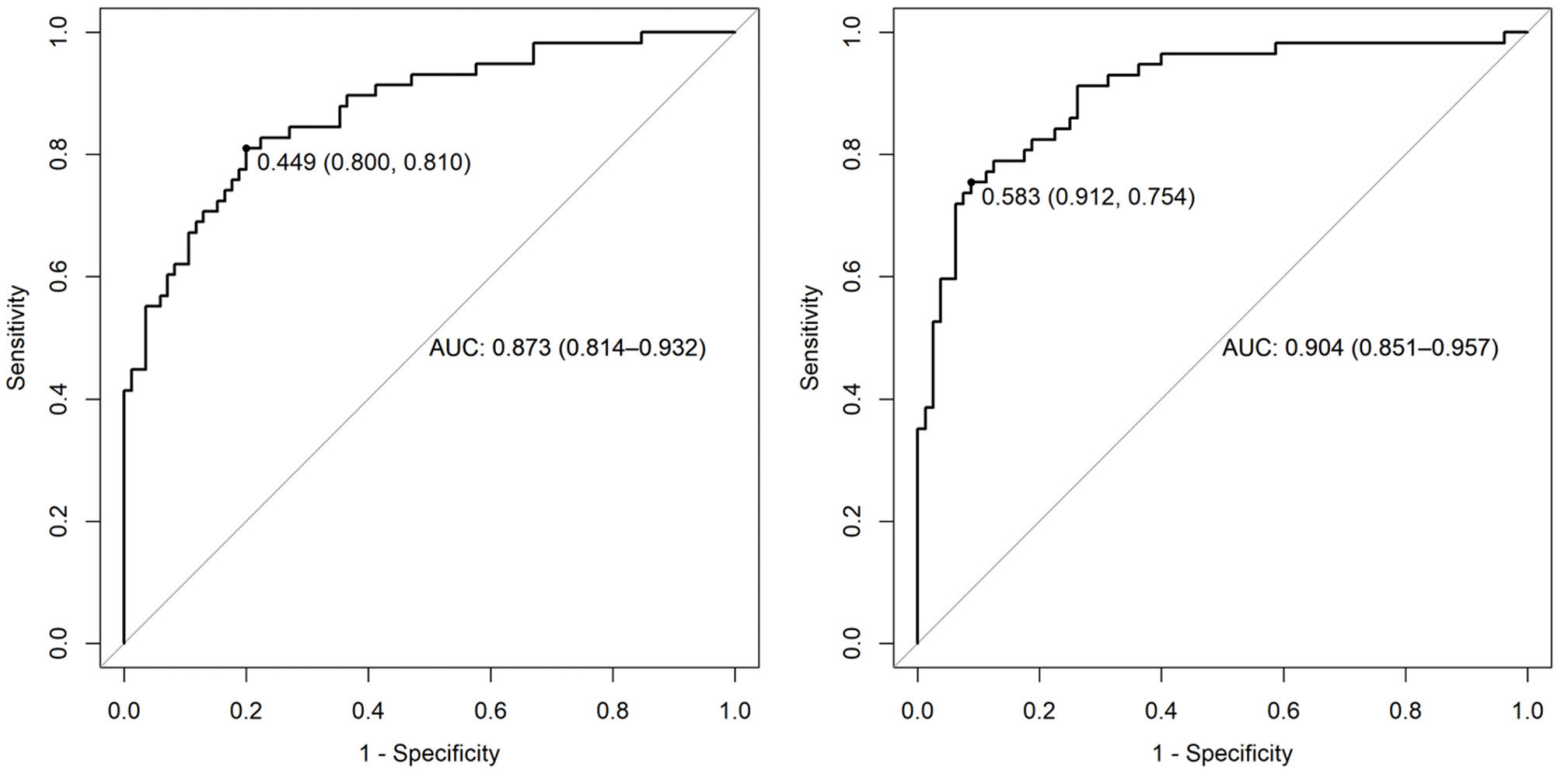
AUC of ROC analysis for prediction of survival. Left panel: variables of the MAGGIC score; right panel: MAGGIC variables and PAC-GTN.

**Figure 4:**
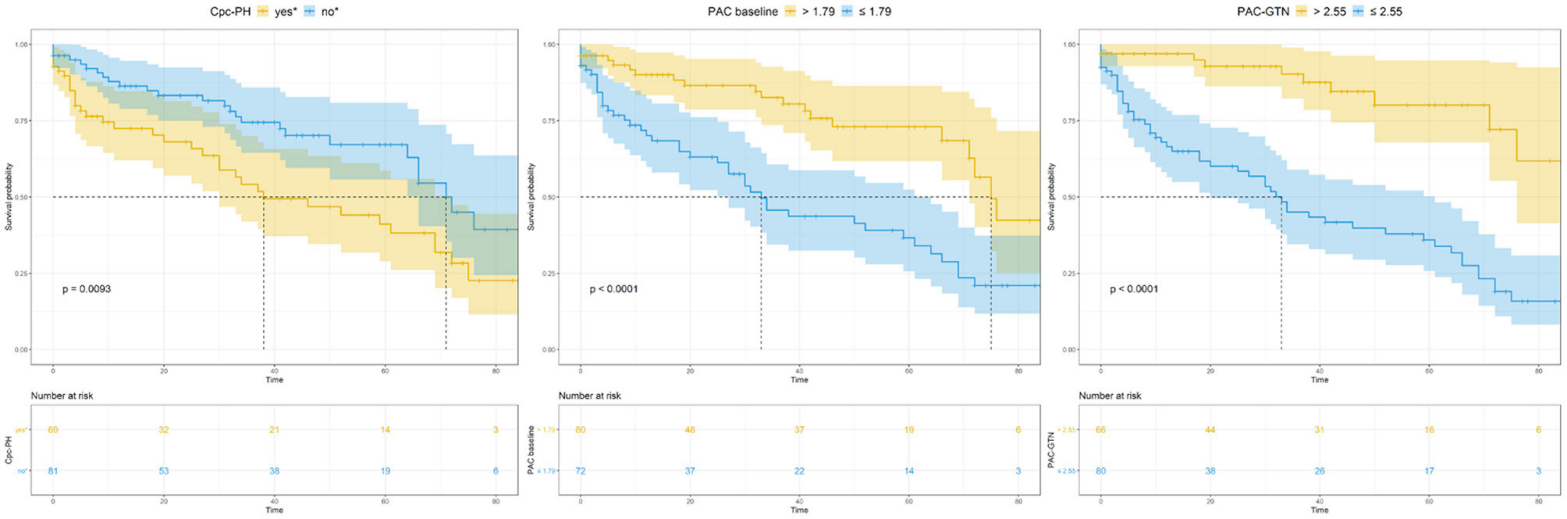
Kaplan-Meier analysis of key variables CpcPH, baseline PAC, and PAC-GTN. CpcPH, combined post- and pre-capillary pulmonary hypertension (“yes,” if pulmonary vascular resistance >3 wood units and/or diastolic pulmonary gradient ≥7 mmHg); PAC, pulmonary arterial compliance; GTN, glyceryl trinitrate.

This significant finding was the basis for further analyses. PAC-GTN was able to improve the AUC in the ROC analysis of the MAGGIC score to differentiate patients with an unfavorable outcome (Figure 3). PAC-GTN and delta PAC were not correlated to the GTN dose administered (Spearman *r* = −0.14; *p* = 0.09, and *r* = −0.04; *p* = 0.60). Kaplan-Meier survival analyses confirmed the prognostic power of PAC-GTN. Although presence of CpcPH and baseline PAC were both associated with survival, PAC-GTN was superior in prognostication (Figure 4). Kaplan-Meier subgroup analysis in patients with HFpEF and in patients with HFmrEF/HFrEF demonstrated that PAC-GTN was associated with survival in both groups, and the association appeared stronger in HFpEF patients (Supplementary Figures 2, 3). Delta PAC and percentage increase in PAC after GTN administration were also significantly associated with survival (delta PAC: cutoff 0.52 ml/mmHg, *p* <0.0001; percentage increase in PAC: cutoff 0.23%, *p* <0.0001; Supplementary Figures 4, 5).

Interestingly, reduction of RV oscillatory power and thus oscillatory load was more pronounced in PAC-GTN >2.55 than ≤ 2.55 ml/mmHg (median −0.025 vs. −0.010 W; *p* <0.0001), whereas the extent of PVR reduction was even smaller in PAC-GTN >2.55 than ≤ 2.55 ml/mmHg (-0.39 vs.−0.78 WU; *p* = 0.031).

To explore whether classification according to PAC-GTN instead of CpcPH would lead to a significant change in prognostication, we performed a reclassification analysis. Seventy-one patients were classified as high risk according to CpcPH criteria; they all had PVR >3 WU, and 8 of them additionally had DPG ≥ 7 mmHg. Hence, DPG did not influence risk stratification. Fourteen of those patients with PVR >3 WU had PAC-GTN > 2.55 ml/mmHg (thus changing from high to low risk), and 24 patients with PVR ≤ 3 WU had PAC-GTN ≤ 2.55 ml/mmHg (thus changing from low to high risk). All in all, 38 patients (25%) had different hemodynamic prognostication by either PVR (CpcPH) or PAC-GTN (cut-off).

## Discussion

Here we present a comprehensive analysis of the prognostic value of invasive hemodynamics at baseline and after challenge with sublingual GTN in HF patients with post-capillary PH. The relevant findings of our study are as follows: (i) three hemodynamic parameters (PAC, Ea, TPR) obtained after administration of GTN, all of them derived from pressure/flow relationships, showed significant prognostic value; (ii) PAC-GTN was the best prognostic marker, which was superior to established parameters such as PAC (19) and the presence of CpcPH; (iii) PAC-GTN may be a surrogate for a successful reduction of RV oscillatory load.

There are few reports available concerning prognostic implications of VRT in pre-capillary PH (20, 21). In candidates for cardiac transplantation, reversibility of post-capillary PH and thus a better outcome post-transplantation is assumed if TPG decreases to <15 mmHg and/or pulmonary vascular resistance decreases to <3 WU. In other heart failure patients, VRT is currently not recommended, and a consistent protocol is lacking as well as the definition of a positive test result (22, 23). Ghio et al. (24) conducted VRT using intravenous nitrates in 156 heart failure patients with a reduced LVEF and PH and found that survival was significantly reduced in non-responders in contrast to responders. In a study by Al-Naamani et al. (25) VRT did not predict outcome in 73 patients with PH and heart failure with preserved LVEF. Lim et al. (26) described an association of PVR reduction (at least 20%) with survival, and baseline PAC was associated with survival in 98 patients with “mixed” PH. To the best of our knowledge, however, our study is the first to demonstrate a prognostic value of VRT in post-capillary PH independent of the “CpcPH” definition and a predefined, albeit arbitrary definition of “response.”

Three factors may have contributed to these new findings: the vasodilator used and its dosage, the measurement methods, and the most suitable hemodynamic parameter. Most drugs used for VRT are more or less selective pulmonary vasodilators that cause a small decrease or even an increase in PAWP, which is undesired in LHF (but nevertheless may result in PVR reduction). GTN decreases PAP and PAWP markedly by provoking venous and also arterial vasodilation, thus lowering pre- and afterload and indirectly increasing subendocardial blood flow (11, 27). Therefore, GTN causes much more than pulmonary vasodilation: the whole RV-PA-LV unit is unloaded in a dose-dependent manner. The term “unloading test” would describe these combined effects better than “vasoreactivity test.” However, the primary component contributing to improved PAC by GTN in the survivors of our cohort was the decrease in PA pulse pressure rather than an increase in stroke volume. In line with this, PAPi as an index of right ventricular contractility independent of CO measurement (28) did not show prognostic relevance. Pulse pressure after GTN alone was also prognostic, but weaker than PAC-GTN. In our cohort, patients with preserved LVEF showed a larger reduction of pulse pressure in response to GTN than those with reduced LVEF, but a smaller increase of cardiac output.

Single hemodynamic measurements may be subject to the bias of situational influences such as vasoconstriction and may mitigate the prognostic power of established hemodynamic indices such as CpcPH. Repeated measurements after vasodilatory challenge and thus ventricular unloading may be advantageous in this context. If unloading does not lead to markedly improved pressure-flow relationships (which are the basis for calculation of the abovementioned three key variables), structural pulmonary vasculopathy may be present.

Our analysis took multiple established prognostic hemodynamic factors into account, and Ea, TPR, and PAC as indicators of RV afterload (29) measured after GTN challenge showed the best associations with prognosis. Among them, PAC-GTN stood out and yielded a clear cut-off value. PAC may be superior to PVR because it “bundles the effects of PVR and left-sided filling pressures on RV afterload.” (19) Furthermore, PAC integrates resistive, pulsatile, and passive components of RV afterload and therefore may reflect remodeling of the PA (30). Reduction of the RV oscilllatory load is likely to be the dominant effect of PAC increase in our cohort, for the fall of RV oscilllatory load was markedly more pronounced in the PAC-GTN >2.55 group, and reduction of the steady component of RV load (delta PVR) was relatively weak. RV dysfunction or RV-PA coupling may be better defined by Ea and PAC than by other parameters (29). However, cut-off values proposed for PAC as a risk marker vary widely (19, 31, 32); repeated measurements after vasodilatory challenge could be a method to obtain a more consistent cut-off value.

### Limitations

We included patients with different types of heart failure (HFpEF, HFmrEF and HFrEF), which may be a source of bias. However, the effects of all types are elevated left-sided filling pressures, resulting in post-capillary PH (8). Our analysis of patients with HFpEF vs. HFmrEF/HFrEF confirmed our main results in both groups. Furthermore, 23 patients (12, 5 % of the cohort assessed for eligibility) were lost to follow up, which seems to be within an acceptable range (33).

### Conclusions

A hemodynamic unloading test using GTN may improve the prognostic power of PAC in patients with post-capillary PH and should be investigated in further prospective studies. Implications for therapeutic options of patients defined as high risk by this method remain elusive.

## Data Availability Statement

The original contributions presented in the study are included in the article/Supplementary Materials, further inquiries can be directed to the corresponding author.

## Ethics Statement

The studies involving human participants were reviewed and approved by Ethics Committee of the Faculty of Medicine at the University of Giessen. The patients/participants provided their written informed consent to participate in this study.

## Author Contributions

AR, DG, and TK: conception and design, statistical analysis, interpretation of data, and drafting of the manuscript, GZ: acquisition of data. SK, SW, MR, KT, UK, VM, CH, and SR: analysis and interpretation of data and revising the manuscript critically for important intellectual content. All authors: final approval of the manuscript submitted.

## Funding

This research project uses a cohort that is part of the Kerckhoff Biomarker Registry (BioReg), which is financially supported by the Kerckhoff Heart Research Institute (KHFI) and the German Center for Cardiovascular Research e.V. (DZHK). The sponsors had no influence on the study design, statistical analyses, or draft of the article.

## Conflict of Interest

The authors declare that the research was conducted in the absence of any commercial or financial relationships that could be construed as a potential conflict of interest.

## Publisher's Note

All claims expressed in this article are solely those of the authors and do not necessarily represent those of their affiliated organizations, or those of the publisher, the editors and the reviewers. Any product that may be evaluated in this article, or claim that may be made by its manufacturer, is not guaranteed or endorsed by the publisher.

## Abbreviations

PH: pulmonary hypertension
HF: heart failure
RHC: right heart catheterisation
VRT: vasoreactivity testing
GTN: glyceryl trinitrate
LHF: left heart failure
PVR: pulmonary vascular resistance
PAC: pulmonary arterial compliance
DPG: diastolic pressure gradient
CpcPH: combined post- and pre-capillary pulmonary hypertension
IpcPH: isolated post-capillary pulmonary hypertension
TD: thermodilution
SV: stroke volume
TPR: total pulmonary resistance
PA: pulmonary arterial
PP: pulmonary arterial pulse pressure
Ea: effective PA elastance
PAPi: PA pulsatility index
RV: right ventricular
MAGGIC: Meta-Analysis Global Group in Chronic Heart Failure.
