# Supplementary material for: Prognostic Power of Pulmonary Arterial Compliance Is Boosted by a Hemodynamic Unloading Test With Glyceryl Trinitrate in Heart Failure Patients With Post-capillary Pulmonary Hypertension
Source: Front Cardiovasc Med. 2022 Mar 31;9:838898. doi: 10.3389/fcvm.2022.838898 (PMC9008270; doi:10.3389/fcvm.2022.838898)
Supplement: Supplementary file 1 [file Table_1.DOCX]

**Supplementary Tables**

**Table S1. Hemodynamics at baseline and after GTN administration**

|  | *Data availability* | Baseline | GTN | p-Value* |
| --- | --- | --- | --- | --- |
|  |  | n = 154 | n = 154 |  |
| Systolic BP, mmHg | *154 / 154* | 127 [109-145] | 117 [104-134] | <0.0001^b^ |
| Mean BP, mmHg | *153 (152) / 154* | 89 [82-101] | 84 [77-93] | <0.0001^b^ |
| Heart rate, beats/min | *154 (153) / 154* | 67 [62-74] | 68 [61-74] | 0.1449^b^ |
| PAWP, mmHg | *153 / 154* | 23.7 (±5.5) | 17.0 [12.0-21.0] | <0.0001^b^ |
| sPAP, mmHg | *154 / 154* | 58.0 [49.0-70.0] | 47.0 [34.3-57.8] | <0.000^b^ |
| mPAP, mmHg | *154 / 154* | 37.1 (±7.7) | 28.3 (±8.1) | <0.0001^a^ |
| dPAP, mmHg | *154 / 154* | 22.0 [18.0-26.0] | 17.3 (±5.6) | <0.0001^b^ |
| PP, mmHg | *154 / 154* | 35.0 [28.0-43.0] | 27.5 [19.3-37.0] | <0.0001^b^ |
| RAP, mmHg | *154 (136) / 154* | 11.0 [8.0-15.0] | 8.0 [5.0-11.0] | <0.0001^b^ |
| TPG, mmHg | *153 / 154* | 12.0 [9.0-17.0] | 12.0 [8.0-15.0] | <0.0001^b^ |
| DPG, mmHg | *153 / 154* | −1.0 [−4.0-2.0] | 1.0 [−2.0-4.0] | <0.0001^b^ |
| CO-TD, l/min | *154 (149) / 154* | 4.36 [3.53-5.29] | 4.70 [3.80-5.63] | <0.0001^b^ |
| CI-TD, l/min/m^2^ | *154 (149) / 154* | 2.21 [1.93-2.64] | 2.33 [2.01-2.80] | <0.0001^b^ |
| SV-TD, mL | *154 (148) / 154* | 64.13 [50.95-78.02] | 67.81 [53.70-84.04] | <0.0001^b^ |
| PVR, WU | *152 (148) / 154* | 2.90 [2.02-4.09] | 2.31 [1.64-3.62] | <0.0001^b^ |
| SVR, WU | *153 (133) /154* | 18.25 [14.69-23.33] | 15.71 [12.42-20.29] | <0.0001^b^ |
| PVR/SVR | *151 (132) /154* | 0.16 [0.12-0.21] | 0.15 [0.11-0.22] | 0.0724^b^ |
| TPR, WU | *154 (149) / 154* | 8.38 [6.55-10.79] | 5.83 [4.58-7.69] | <0.0001^b^ |
| PAC, mL/mmHg | *154 (148) / 154* | 1.84 [1.33-2.35] | 2.45 [1.77-3.48] | <0.0001^b^ |
| Ea, mmHg/mL | *154 (148) / 154* | 0.85 [0.60-1.07] | 0.57 [0.39-0.76] | <0.0001^b^ |
| PAPi | *154 (136) / 154* | 3.10 [2.22-4.32] | 3.44 [2.63-5.00] | <0.0001^b^ |
| RAP/PAWP | *152 (135) / 154* | 0.52 [0.39-0.62] | 0.50 [0.40-0.60] | 0.0032^b^ |
| RV power_oscill_, W | *154 (149) / 154* | 0.10 [0.08-0.13] | 0.08 [0.07-0.11] | <0.0001^b^ |

Data are displayed as median [interquartile range] or mean (± standard deviation) except where otherwise indicated. Data availability: numbers after GTN administration are given in brackets, if different from baseline.

GTN, glycerol trinitrate; BP, blood pressure; PAWP, pulmonary arterial wedge pressure;

sPAP, systolic pulmonary arterial pressure; mPAP, mean PAP; dPAP, diastolic PAP; PP, pulse pressure; RAP: mean right atrial pressure; TPG, transpulmonary gradient; DPG, diastolic pulmonary gradient; CO, cardiac output; TD, Thermodilution method; CI, cardiac index; SV, stroke volume; PVR, pulmonary vascular resistance; WU, Wood units; SVR, systemic vascular resistance; TPR, total pulmonary resistance; PAC, pulmonary arterial compliance; Ea, pulmonary effective arterial elastance (calculated); PAPi, pulmonary artery pulsatility index; RV, right ventricle; oscill, oscillatory; W, watt.

*Baseline vs GTN. ^a^Student´s t-test. ^b^Wilcoxon signed rank test.

**Table S2. Hemodynamics in different heart failure types**

|  | *Data availability* | All | HFpEF (1)  N=74 | HFmrEF (2)  N=12 | HFrEF (3)  N=68 | p-  p [1vs.2] | Value*  p [1vs.3] | p [2vs.3] |
| --- | --- | --- | --- | --- | --- | --- | --- | --- |
| GTN dose, mg | *151 / 154* | 2.4 [2-3.2] | 2.8 [2.4-3.2] | 2 [1.2-2.4] | 2.4 [2.4-3.2] | 0.028 | 0.800 | 0.029 |
| Systolic BP, mmHg | *154 / 154* | 127[109-145] | 136 [120-154] | 129 [114-156] | 113 [103-129] | 0.421 | <0.001 | 0.051 |
| Systolic BP-GTN, mmHg | *154 / 154* | 117 [104-134] | 127 [114-142] | 118.5 [109-137] | 106 [101-118] | 0.418 | <0.001 | 0.051 |
| Delta Systolic BP, mmHg | *154 / 154* | −9 [−17-−2] | −12 [−18-−4] | −13 [−17-−1] | −6 [−13-−1] | 0.871 | 0.013 | 0.257 |
| Mean BP, mmHg | *153 / 154* | 89 [82-101] | 93 [87-108] | 95 [86-109] | 85 [79-93] | 0.839 | <0.001 | 0.026 |
| Mean BP-GTN, mmHg | *152 / 154* | 84 [77-93] | 88 [81-96] | 89 [81-97] | 79 [73-85] | 0.990 | <0.001 | 0.049 |
| Delta Mean BP, mmHg | *152 / 154* | −6 [−13-−1] | −6 [−17-−1] | −12 [−15-−1] | −6 [-11-−1] | 0.569 | 0.520 | 0.236 |
| Heart rate, beats/min | *154 / 154* | 67 [62-74] | 66 [62-75] | 70,5 [64-73] | 68.5 [63-72] | 0.896 | 0.893 | 0.829 |
| Heart rate-GTN, beats/min | *153 / 154* | 68 [61-74] | 67 [61-75] | 71 [64-74] | 69 [61-73] | 0.762 | 0.929 | 0.627 |
| Delta Heart rate, beats/min | *153 / 154* | 0 [−2-3] | 1 [−1-3] | 2 [−4-4] | 0 [−2-2] | 0.589 | 0.636 | 0.551 |
| PAWP, mmHg | *152 / 154* | 23.7 (± 5.5) | 22.3 (± 5.1) | 21.7 (± 4.3) | (25.5 ± 5.7) | 0.629 | 0.001 | 0.013 |
| PAWP-GTN, mmHg | *153 / 154* | 17.0 [12.0-21.0] | 15.0 [11.3-19.0] | 17.5 [12.8-21.8] | 18.0 [13.0-22.0] | 0.339 | 0.008 | 0.758 |
| Delta PAWP, mmHg | *152 / 154* | −6.0 [−10.0-−3.0] | −6.0 [−9.8-−4.0] | −4.5 [−6.3-−3.0] | −6.0 [−12.8-−3.0] | 0.092 | 0.725 | 0.115 |
| mPAP, mmHg | *154 / 154* | 37.1 (± 7.7) | 36.5 (± 8.3) | 34.5 (± 6.3) | 38.1 (± 7.2) | 0.339 | 0.231 | 0.093 |
| mPAP-GTN, mmHg | *154 / 154* | 28.3 (± 8.08) | 27.1 (± 8.6) | 26.5 (± 7.8) | 29.8 (± 7.4) | 0.811 | 0.045 | 0.190 |
| Delta mPAP, mmHg | *154 / 154* | −7.0 [−12.0-−5.0] | −8.0 [−11.8-−5.0] | −7.5 [−10.0-−4.0] | −7.0 [−12.0-−4.8] | 0.439 | 0.230 | 0.903 |
| sPAP, mmHg | *154 / 154* | 58.0 [49.0-70.0] | 57.5 [47.3-70.0] | 54.5 [48.0-64.0] | 58.5 [52.0-70.0] | 0.704 | 0.328 | 0.287 |
| sPAP-GTN, mmHg | *154 / 154* | 47.0 [34.3-57.8] | 40.0 [33.0-57.5] | 44.5 [34.0-52.0] | 49.5 [37.8-58.0] | 0.955 | 0.072 | 0.215 |
| Delta sPAP, mmHg | *154 / 154* | −10.0 [−18.0-−5.3] | −13.0 [−18.0-−7.3] | −9.0 [−19.0-−5.8] | −8.0 [−18.0-−4.8] | 0.525 | 0.071 | 0.637 |
| dPAP, mm | *154 / 154* | 22.0 [18.0-26.0] | 22.0 [17.0-25.0] | 20.0 [17.8-24.5] | 23.5 [20.0-27.0] | 0.886 | 0.027 | 0.162 |
| dPAP-GTN, mm | *154 / 154* | 17.3 (± 5.63) | 16.6 (± 5.6) | 16.8 (± 5.8) | 18.3 (± 5.5) | 0.915 | 0.073 | 0.421 |
| Delta dPAP, mmHg | *154 / 154* | −5.0 [−7.0-−3.0] | −5.0 [−7.0-−3.0] | −6.0 [−9.0-−2.0] | −6.0 [−7.0-−2.0] | 0.680 | 0.524 | 0.957 |
| TPG, mmHg | *152 / 154* | 12.0 [9.0-17.0] | 14.0 [9.0-18.0] | 12.0 [10.0-14.5] | 12.0 [9.0-15.0] | 0.694 | 0.290 | 0.760 |
| TPG-GTN, mmHg | *153 / 154* | 12.0 [8.0-15.0] | 11.5 [8.0-15.0] | 9.0 [6.8-11.3] | 12.0 [9.0-15.0] | 0.104 | 0.454 | 0.021 |
| Delta TPG, mmHg | *152 / 154* | −1.7 (± 4.3) | −2.4 (± 4.2) | −3.6 (± 3.3) | −0.6 (± 4.2) | 0.281 | 0.012 | 0.013 |
| DPG, mmHg | *152 / 154* | −1.0 [−4.0-2.0] | -1.0 [−4.0-3.0] | −1.0 [−2.0-2.5] | −1.0 [−4.0-1.0] | 0.662 | 0.429 | 0.389 |
| DPG-GTN, mmHg | *153 / 154* | 1.0 [−2.0-4.0] | 1.5 [−1.0-5.0] | −0.5 [−2.3-1.8] | 2.0 [−2.5-4.0] | 0.202 | 0.656 | 0.338 |
| Delta DPG, mmHg | *152 / 154* | 1.8 (± 4.9) | 1.9 (± 4.2) | −0.3 (± 4.2) | 2.1 (± 5.6) | 0.116 | 0.880 | 0.114 |
| PP, mmHg | *154 / 154* | 35.0 [28.0-43.0] | 35.5 [28.0-44.8] | 31.5 [27.8-39.8] | 35.0 [28.0-43.0] | 0.694 | 0.951 | 0.590 |
| PP-GTN, mmHg | *154 / 154* | 27.5 [19.3-37.0] | 25 [18.3-36.8] | 23.5 [21.3-34.3] | 31 [23.5-37.3] | 0.940 | 0.111 | 0.305 |
| Delta PP, mmHg | *154 / 154* | −6.5 [−13.0-−1.0] | −7.5 [−13.0-−4.3] | −6.5 [−11.5-−3.5] | −4.0 [−11.3−1.0] | 0.626 | 0.014 | 0.322 |
| RAP, mmHg | *154 / 154* | 11.0 [8.0-15.0] | 11.0 [9.0-14.8] | 13.0 [6.5-15.3] | 12.0 [6.8-16.0] | 0.886 | 0.868 | 0.984 |
| RAP-GTN, mmHg N | *136 / 154* | 8.0 [5.0-11.0] | 8.0 [5.0-10.0] | 9.0 [5.0-11.5] | 9.0 [5.0-12.0] | 0.630 | 0.690 | 0.859 |
| Delta RAP, mmHg | *136 / 154* | −3.5 [−5.0-−2.0] | −3.0 [−4.0-−2.3] | −2.0 [−3.5-−0.5] | −4.0 [−5.0-−2.0] | 0.034 | 0.687 | 0.037 |
| CO-TD, l/min e | *154 / 154* | 4.36 [3.53-5.29] | 4.70 [4.01-5.68] | 4.23 [3.71-5.51] | 3.75 [3.19-4.52] | 0.166 | <0.001 | 0.147 |
| CO-TD-GTN, l/min | *149 / 154* | 4.70 [3.80-5.63] | 4.95 [4.08-5.95] | 4.95 [4.04-5.91] | 4.25 [3.65-5.2] | 0.851 | 0.003 | 0.172 |
| Delta CO-TD, l/min | *149 / 154* | 0.37 (± 0.66) | 0.19 (± 0.68) | 0.64 (± 0.70) | 0.52 (± 0.59) | 0.057 | 0.004 | 0.559 |
| CI-TD, l/min/m^2^ | *154 / 154* | 2.21 [1.93-2.64] | 2.55 [2.17-2.79] | 2.27 [2.03-2.52] | 1.96 [1.64-2.29] | 0.090 | <0.001 | 0.028 |
| CI-TD-GTN, l/min/m^2^ | *149 / 154* | 2.33 [2.01-2.80] | 2.54 [2.18-3.04] | 2.59 [2.11-2.97] | 2.06 [1.89-2.55] | 0.831 | <0.001 | 0.024 |
| Delta CI-TD, l/min/m^2^ | *149 / 154* | 0.19 (± 0.33) | 0.10 (± 0.35) | 0.32(± 0.34) | 0.25 (± 0.29) | 0.058 | 0.005 | 0.560 |
| SV-TD, mL | *154 / 154* | 64.13 [50.95-78.02] | 70.09 [61.25-83.90] | 66.28 [52.38-76.76] | 55.41 [45.27-69.01] | 0.224 | <0.001 | 0.194 |
| SV-TD-GTN, mL | *148 / 154* | 67.81 [53.70-84.04] | 75.68 [61.54-84.98] | 68.22 [59.79-81.76] | 61.00 [51.67-77.92] | 0.595 | 0.007 | 0.228 |
| Delta SV-TD, mL | *148 / 154* | 4.79 (± 10.5) | 2.08 (± 10.61) | 8.20 (± 12.66) | 7.05 (± 9.35) | 0.136 | 0.004 | 0.768 |
| PVR, WU | *152 / 154* | 2.9 [2.0-4.1] | 2.6 [1.8-3.7] | 2.6 [2.2-3.2] | 3.2 [2.2-4.4] | 0.704 | 0.087 | 0.426 |
| PVR-GTN, WU | *148 / 154* | 2.3 [1.6-3.6] | 2.2 [1.6-3.0] | 1.7 [1.2-3.0] | 2.56 [2.0-3.7] | 0.224 | 0.039 | 0.027 |
| Delta PVR.,WU | *147 / 154* | −0.7 [−1.1-−0.0] | −0.6 [−1.0-−0.1] | −1.0 [−1.5-−0.5] | −0.6 [−1.2-0.2] | 0.075 | 0.900 | 0.165 |
| SVR, WU | *153 / 154* | 18.3 [14.7-23.3] | 17.6 [13.8-20.8] | 17. [15.4-26.04] | 19.5 [15.2-24.5] | 0.402 | 0.072 | 0.915 |
| SVR-GTN, WU | *133 / 154* | 15.7 [12.4-20.3] | 15.5 [12.7-20.0] | 15.9 [11.7-22.1] | 16.5 [12.5-20.7] | 0.691 | 0.929 | 0.729 |
| Delta SVR, WU | *133 / 154* | −2.2 [−4.1-−0.2] | −1.5 [−3.1-0.3] | −3.5 [−5.5-−2.5] | −2.5 [−4.9-−0.8] | 0.015 | 0.012 | 0.319 |
| PVR/SVR | *151 / 154* | 0.16 [0.12-0.21] | 0.16 [0.11-0.25] | 0.15 [0.14-0.17] | 0.16 [0.13-0.22] | 0.793 | 0.635 | 0.646 |
| PVR/SVR-GTN | *132 / 154* | 0.15 [0.11-0.22] | 0.13 [0.10-0.20] | 0.11 [0.09-0.16] | 0.17 [0.13-0.23] | 0.307 | 0.019 | 0.017 |
| Delta PVR/SVR | *132 / 154* | −0.01 [−0.05-0.02] | −0.02 [−0.06-0.01] | −0.03 [−0.06-0.01] | 0.00 [−0.03-0.03] | 0.731 | 0.097 | 0.245 |
| TPR, WU | *154 / 154* | 8.4 [6.6-10.8] | 6.9 [6.2-9.4] | 7.1 [6.6-8.8] | 9.9 [8.1-12.2] | 0.654 | <0.001 | 0.025 |
| TPR-GTN, WU | *149 / 154* | 5.8 [4.6-7.7] | 5.2 [4.0-6.8] | 4.9 [4.1-6.3] | 7.0 [5.6-8.6] | 0.907 | <0.001 | 0.029 |
| Delta TPR, WU | *149 / 154* | −2.28 [−3.82-−1.37] | −2.00 [-2.66-−1.23] | −2.56 [−2.97-−1.46] | −3.13 [−4.51-−1.61] | 0.164 | 0.001 | 0.293 |
| PAC, mL/mmHg | *154 / 154* | 1.84 [1.33-2.35] | 2.12 [1.46-2.70] | 1.88 [1.59-2.23] | 1.56 [1.26-2.06] | 0.529 | 0.001 | 0.110 |
| PAC-GTN, mL/mmHg | *148 / 154* | 2.45 [1.77-3.48] | 2.83 [2.01-3.58] | 3.03 [1.95-4.18] | 2.12 [1.71-2.59] | 0.743 | 0.002 | 0.091 |
| Delta PAC, mL/mmHg | *148 / 154* | 0.61 [0.23-1.19] | 0.79 [0.30-1.35] | 1.10 [0.55-1.85] | 0.45 [0.17-0.80] | 0.341 | 0.011 | 0.029 |
| Ea, mmHg/mL | *154 / 154* | 0.85 [0.60-1.07] | 0.68 [0.57-0.98] | 0.68 [0.59-0.92] | 0.96 [0.78-1.24] | 0.741 | <0.001 | 0.057 |
| Ea-GTN, mmHg/mL | *148 / 154* | 0.57 [0.39-0.76] | 0.47 [0.34-0.62] | 0.50 [0.34-0.59] | 0.65 [0.48-0.84] | 0.984 | <0.001 | 0.040 |
| Delta Ea, mmHg/mL | *148 / 154* | −0.26 [−0.40-−0.16] | −0.23 [−0.32-−0.15] | −0.29 [−0.34-−0.20] | −0.30 [−0.48-−0.16] | 0.355 | 0.016 | 0.556 |
| PAPi | *154 / 154* | 3.10 [2.22-4.32] | 3.13 [2.39-3.96] | 2.93 [2.38-5.44] | 3.15 [2.20-4.66] | 0.975 | 0.928 | 0.840 |
| PAPi-GTN | *136 / 154* | 3.44 [2.63-5.00] | 3.38 [2.76-4.60] | 2.63 [2.28-5.80] | 3.92 [2.76-5.29] | 0.374 | 0.240 | 0.239 |
| Delta PAPi | *136 / 154* | 0.54 [−0.10-1.44] | 0.48 [−0.22-1.18] | −0.04 [−0.64-1.61] | 1.20 [0.16-2.08] | 0.575 | 0.013 | 0.197 |
| RAP/PAWP | *152 / 154* | 0.52 [0.39-0.62] | 0.54 [0.43-0.61] | 0.56 [0.38-0.64] | 0.44 [0.28-0.64] | 0.955 | 0.132 | 0.542 |
| RAP/PAWP-GTN | 135 / 154 | 0.50 [0.40-0.60] | 0.54 [0.43-0.62] | 0.55 [0.42-0.57] | 0.45 [0.31-0.59] | 0.732 | 0.077 | 0.572 |
| Delta RAP/PAWP | 135 / 154 | −0.02 [−0.09-0.03] | −0.02 [−0.07-0.03] | −0.04 [−0.07-0.05] | −0.02 [−0.13-0.04] | 0.977 | 0.294 | 0.470 |

Data are displayed as median [interquartile range] or mean (± standard deviation) except where otherwise indicated.

HFpEF, heart failure with preserved ejection fraction; HFmrEF, heart failure with mid-range ejection fraction; HFrEF, heart failure with reduced ejection fraction; GTN, glycerol trinitrate; -GTN, values after GTN administration; Delta, difference between baseline and -GTN; BP, blood pressure; PAWP, pulmonary arterial wedge pressure; sPAP, systolic pulmonary arterial pressure; mPAP, mean PAP; dPAP, diastolic PAP; TPG, transpulmonary gradient; DPG, diastolic pulmonary gradient; PP, pulse pressure; RAP, mean right atrial pressure; CO, cardiac output; TD, Thermodilution method; CI, cardiac index; SV, stroke volume; SVR, systemic vascular resistance; WU, Wood units; PVR, pulmonary vascular resistance; TPR, total pulmonary resistance; PAC, pulmonary arterial compliance; Ea, pulmonary effective arterial elastance [calculated]; PAPi, pulmonary artery pulsatility index.

*HFpEF vs HFmrEF, HFpEF vs HFrEF and HFmrEF vs HFrEF as indicated (Student´s t-test for normally distributed parameters or Wilcoxon signed rank test for non-normally distributed parameters)

**Table S3. Univariate regression analysis for death**

|  | unadjusted | | | adjusted | | |
| --- | --- | --- | --- | --- | --- | --- |
|  | OR (CI) per SD increase (z-score) | P-value (OR) | AUC (CI) | OR (CI) per SD increase (z-score) | P-value (OR) | AUC (CI) |
| Ea-GTN | 3.31 (2.03-5.42) | <0.001 | 0.75 (0.68-0.83) | 2.26 (1.30-3.92) | 0.004 | 0.89 (0.83-0.95) |
| PAC-GTN | 0.31 (0.18-0.51) | <0.001 | 0.77 (0.69-0.85) | 0.45 (0.25-0.80) | 0.006 | 0.89 (0.84-0.95) |
| TPR-GTN | 3.33 (2.09-5.30) | <0.001 | 0.77 (0.69-0.84) | 2.29 (1.34-3.93) | 0.003 | 0.89 (0.84-0.95) |
| dPAP | 1.91 (1.33-2.75) | <0.001 | 0.66 (0.58-0.75) | 1.87 (1.14-3.07) | 0.014 | 0.89 (0.83-0.94) |
| sPAP-GTN | 2.00 (1.40-2.88) | <0.001 | 0.69 (0.61-0.78) | 1.86 (1.16-2.97) | 0.010 | 0.89 (0.83-0.94) |
| TAPSE | 0.47 (0.30-0.75) | 0.001 | 0.70 (0.60-0.80) | 0.54 (0.31-0.97) | 0.037 | 0.89 (0.83-0.95) |
| mPAP | 1.72 (1.21-2.43) | 0.003 | 0.65 (0.56-0.74) | 1.78 (1.10-2.87) | 0.019 | 0.89 (0.83-0.94) |
| Delta PAC | 0.43 (0.27-0.68) | <0.001 | 0.73 (0.65-0.81) | 0.51 (0.30-0.88) | 0.014 | 0.89 (0.83-0.95) |
| mPAP-GTN | 2.07 (1.43-2.99) | <0.001 | 0.69 (0.61-0.78) | 1.86 (1.14-3.04) | 0.012 | 0.89 (0.83-0.94) |
| PP-GTN | 1.79 (1.26-2.55) | 0.001 | 0.68 (0.59-0.77) | 1.65 (1.05-2.59) | 0.029 | 0.88 (0.83-0.94) |
| dPAP-GTN | 1.77 (1.25-2.51) | 0.001 | 0.64 (0.56-0.73) | 1.71 (1.06-2.76) | 0.029 | 0.88 (0.83-0.94) |
| Ea | 2.74 (1.78-4.21) | <0.001 | 0.75 (0.67-0.82) | 1.82 (1.13-2.94) | 0.013 | 0.88 (0.83-0.94) |

Adjusted denotes adjustment for the variables of the MAGGIC score.

Ea, pulmonary effective arterial elastance (calculated); GTN, glycerol trinitrate; PAC, pulmonary arterial compliance; TPR, total pulmonary resistance; dPAP, diastolic pulmonary arterial pressure; sPAP, systolic PAP; PAWP, pulmonary arterial wedge pressure; TAPSE, tricuspid annular plane systolic excursion; mPAP, mean PAP; Delta, difference pre / post GTN administration.
