# Supplementary material for: Prognostic Power of Pulmonary Arterial Compliance Is Boosted by a Hemodynamic Unloading Test With Glyceryl Trinitrate in Heart Failure Patients With Post-capillary Pulmonary Hypertension
Source: Front Cardiovasc Med. 2022 Mar 31;9:838898. doi: 10.3389/fcvm.2022.838898 (PMC9008270; doi:10.3389/fcvm.2022.838898)
Supplement: Supplementary file 2 [file Data_Sheet_1.DOCX]

**Supplementary Figures and Legends**


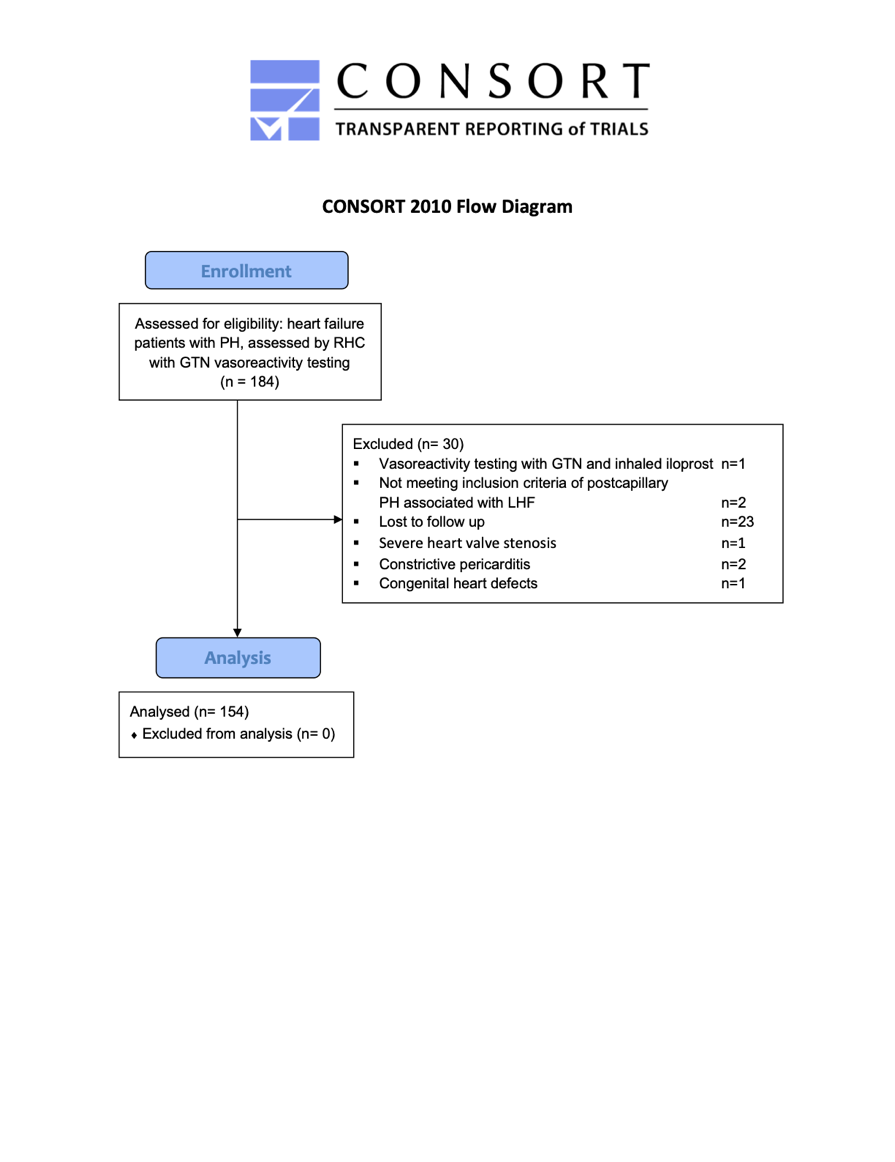


**Figure S1**: Patient flowchart. Abbreviations: PH, pulmonary hypertension; RHC, right heart catheterisation; GTN, glyceryl trinitrate; LHF, left heart failure


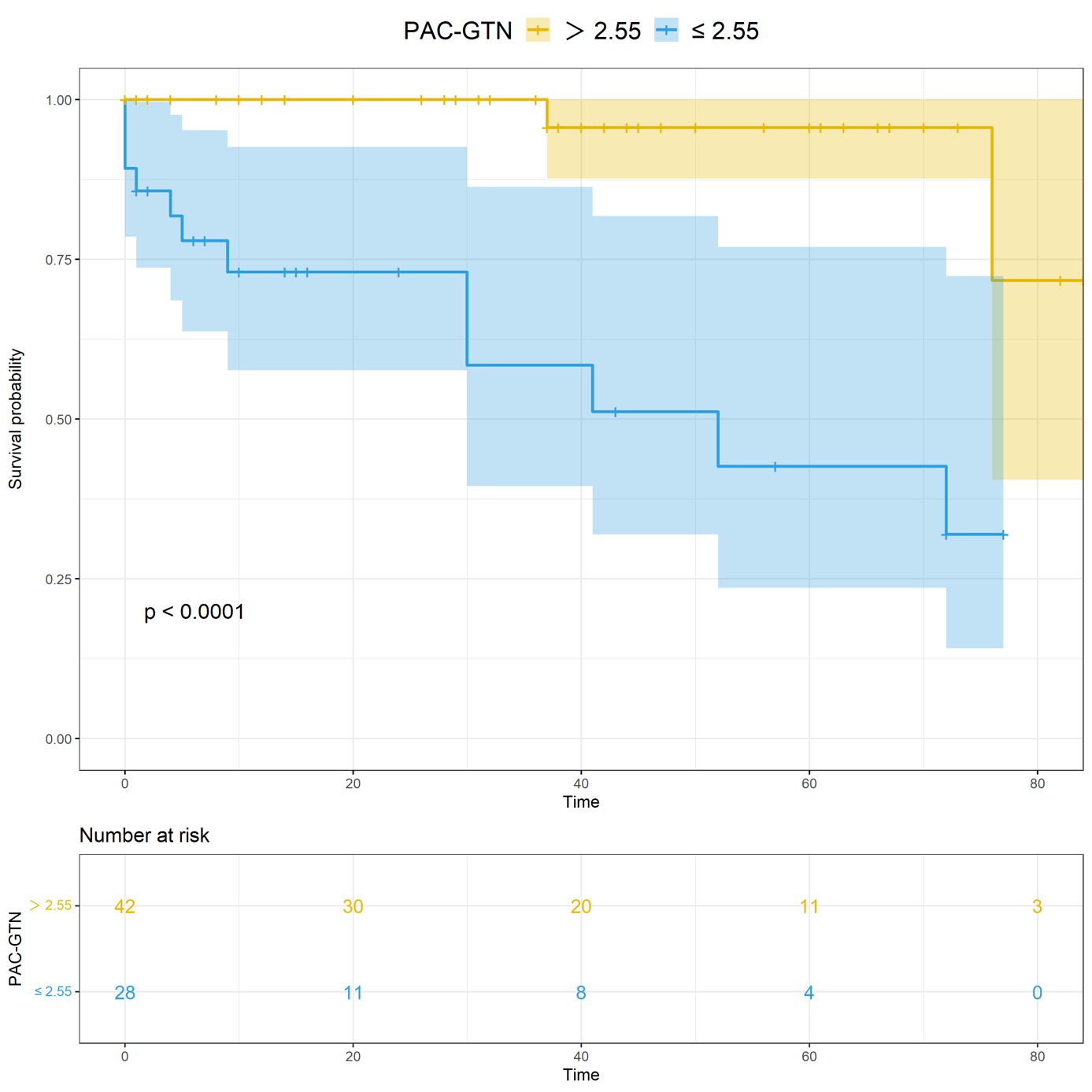


**Figure S2**: Kaplan-Meier analysis of PAC-GTN in patients with HFpEF. Abbreviations: PAC-GTN, pulmonary arterial compliance after administration of glyceryl trinitrate; HFpEF, heart faliure with preserved ejection fraction


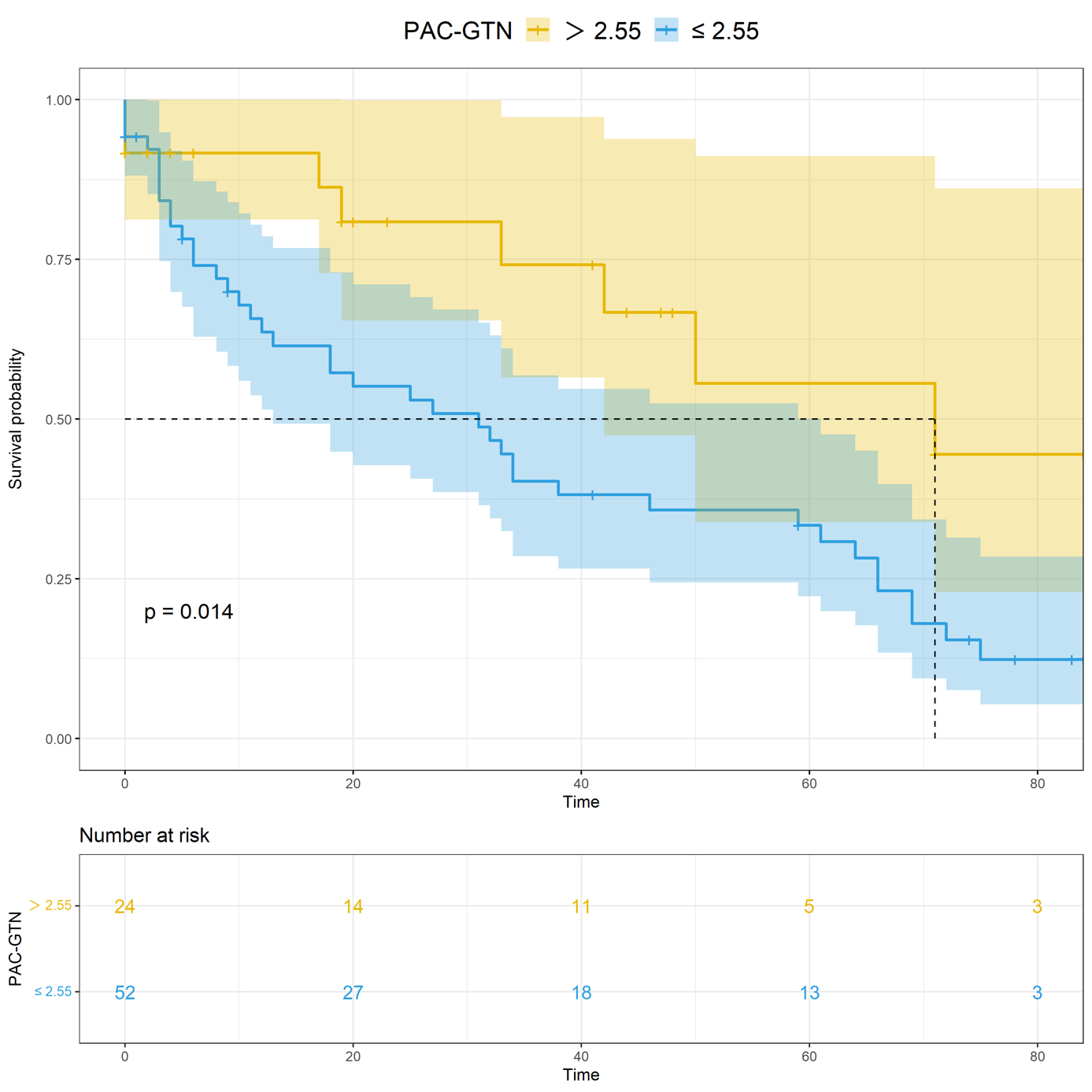


**Figure S3**: Kaplan-Meier analysis of PAC-GTN in patients with HFmrEF and HFrEF. Abbreviations: PAC-GTN, pulmonary arterial compliance after administration of glyceryl trinitrate; HFmrEF, heart faliure with mid range reduced ejection fraction; HFrEF, heart faliure with reduced ejection fraction


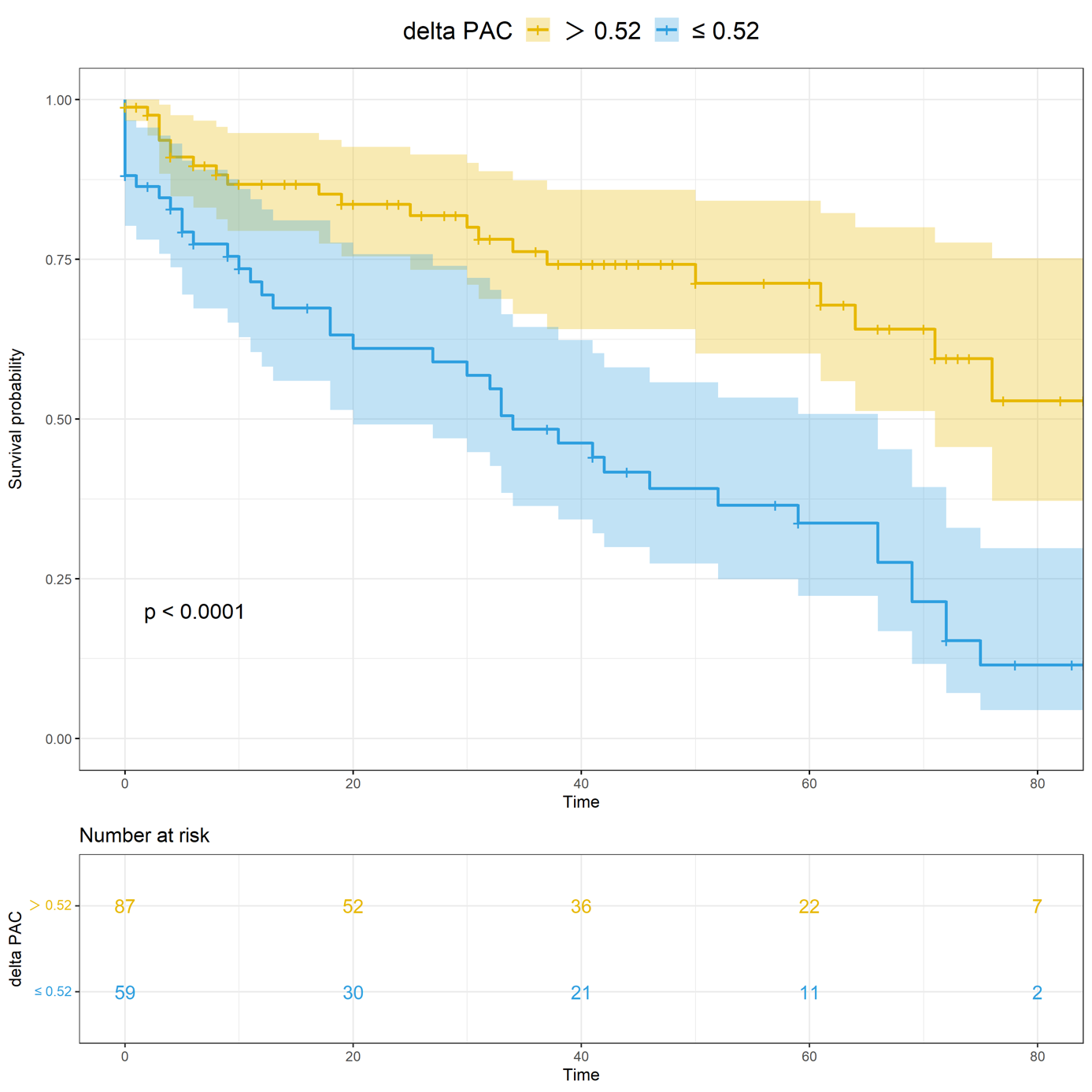


**Figure S4**: Kaplan-Meier analysis of delta PAC in all patients. Abbreviations: PAC, pulmonary arterial compliance; delta, difference between PAC before and after administration of glyceryl trinitrate


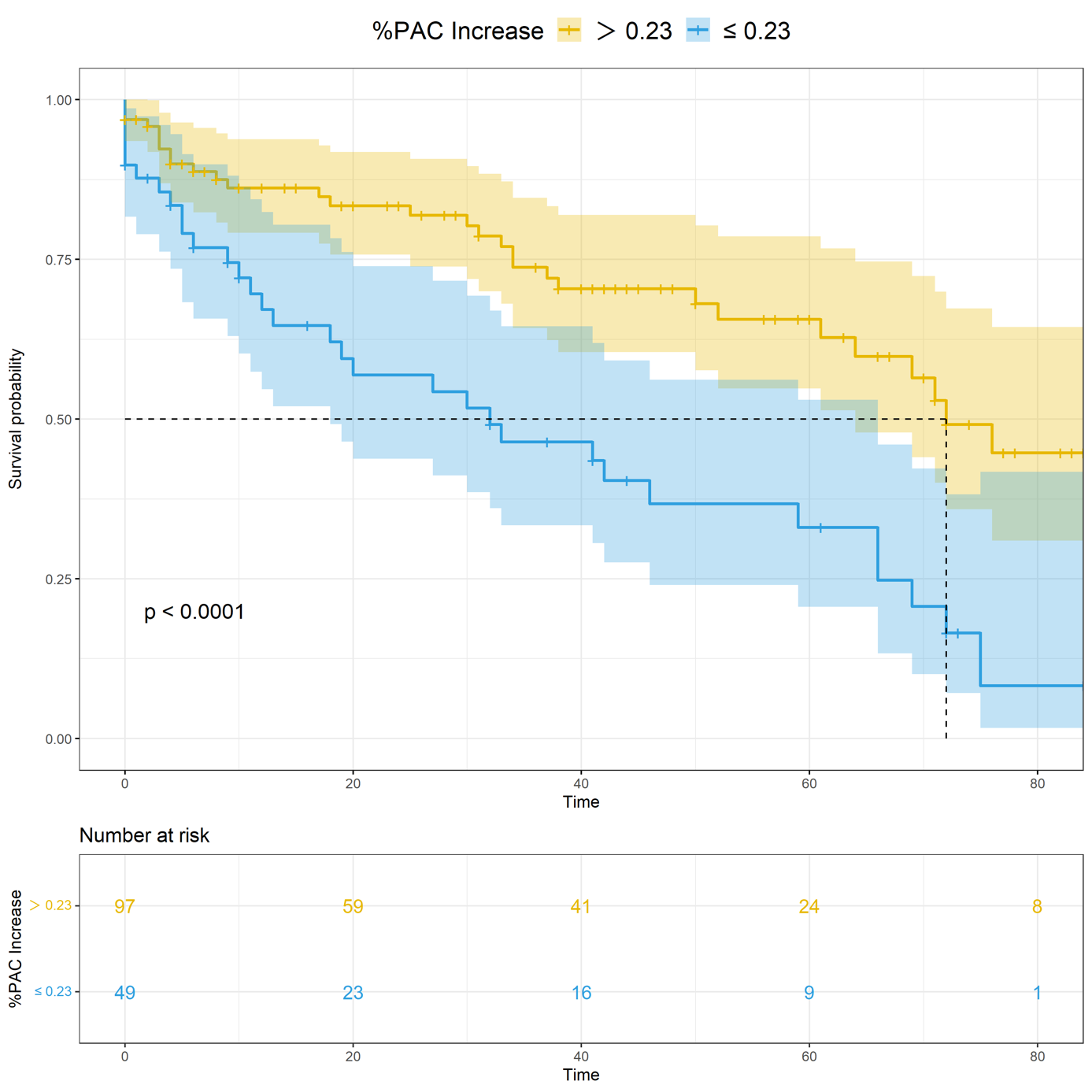


**Figure S5**: Kaplan-Meier analysis of %PAC increase in all patients. Abbreviations: PAC, pulmonary arterial compliance
